# Assessment of Bone Microstructure by Micro CT in C57BL/6J Mice for Sex-Specific Differentiation

**DOI:** 10.3390/ijms232314585

**Published:** 2022-11-23

**Authors:** Katharina Kerschan-Schindl, Maria Papageorgiou, Ursula Föger-Samwald, Maria Butylina, Michael Weber, Peter Pietschmann

**Affiliations:** 1 Department of Physical Medicine, Rehabilitation and Occupational Medicine, Medical University of Vienna, 1090 Vienna, Austria; 2 Institute of Pathophysiology and Allergy Research, Center for Pathophysiology, Infectiology and Immunology, Medical University of Vienna, 1090 Vienna, Austria; 3 Division of Bone Diseases, Geneva University Hospitals and Faculty of Medicine, University of Geneva, 1211 Geneva, Switzerland; 4 Department of Biomedical Imaging and Image-Guided Therapy, Medical University of Vienna, 1090 Vienna, Austria

**Keywords:** microarchitecture, C57BL/6J mice, bone volume/tissue volume, trabecular number, trabecular thickness

## Abstract

It remains uncertain which skeletal sites and parameters should be analyzed in rodent studies evaluating bone health and disease. In this cross-sectional mouse study using micro-computed tomography (µCT), we explored: (1) which microstructural parameters can be used to discriminate female from male bones and (2) whether it is meaningful to evaluate more than one bone site. Microstructural parameters of the trabecular and/or cortical compartments of the femur, tibia, thoracic and lumbar vertebral bodies, and skull were evaluated by µCT in 10 female and 10 male six-month-old C57BL/6J mice. The trabecular number (TbN) was significantly higher, while the trabecular separation (TbSp) was significantly lower in male compared to female mice at all skeletal sites assessed. Overall, bone volume/tissue volume (BV/TV) was also significantly higher in male vs. female mice (except for the thoracic spine, which did not differ by sex). Most parameters of the cortical bone microstructure did not differ between male and female mice. BV/TV, TbN, and TbSp at the femur, and TbN and TbSp at the tibia and lumbar spine could fully (100%) discriminate female from male bones. Cortical thickness (CtTh) at the femur was the best parameter to detect sex differences in the cortical compartment (AUC = 0.914). In 6-month-old C57BL/6J mice, BV/TV, TbN, and TbSp can be used to distinguish male from female bones. Whenever it is not possible to assess multiple bone sites, we propose to evaluate the bone microstructure of the femur for detecting potential sex differences.

## 1. Introduction

Some areas of the skeleton (i.e., hip, spine, wrist, or humerus) are typical for osteoporotic fractures, while others are not. Heterogeneity of bone structure within the trabecular and cortical compartments [1,2] may account for the site-specific differences in fragility fracture risk. Additionally, sex differences in bone microstructure are known [3,4,5,6,7,8,9] and can partially explain the differences in fracture risk between men and women [10].

Animal models, especially inbred mouse strains, provide important insights into skeletal heterogeneity (i.e., local variability in bone properties) and numerous aspects of bone health and disease [11,12,13,14]. Until now, there has been no consensus on which or how many skeletal sites should be investigated in animal studies. Sex differences in bone microstructure have rarely been the primary focus of animal research, while some mouse studies have explored sex-related differences in bone parameters as secondary outcomes [15,16,17]. For example, Glatt et al. [13] described age-related changes in bone microarchitecture in female and male C57BL/6J mice, while detailed data were only provided for the fifth lumbar vertebral body and the distal femur in animals of both sexes at the age of 2 months. Our study aims to give further insights into the sex-specificity of the skeleton by analyzing trabecular and cortical bone microstructure by microcomputed tomography (μCT) in C57BL/6J mice. We purposefully chose this mouse strain as the C57BL/6J mice present with a low bone mass phenotype, and thus, they are frequently used in osteoporosis research and transgenic mouse models. In particular, we first explored which microstructural parameters can be used to discriminate female from male bones. Given that the analysis of bone microstructure by μCT is quite time-consuming, we also aimed to determine if the assessment of one bone site will suffice or whether the evaluation of additional sites could provide complementary information.

## 2. Results

### 2.1. Serum Levels of Bone Turnover Markers

**Figure 1:**
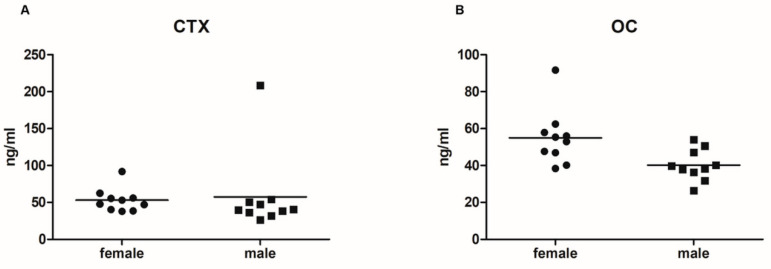
Data points of serum levels of (**A**) C-terminal telopeptide of type I procollagen (CTX) and (**B**) osteocalcin (Oc).

The median serum levels of CTX were 50.30 [39.76; 57.64] ng/mL in female mice and 39.99 [35.18; 51.44] ng/mL in male mice. A single CTX value of a male mouse was extremely high (208.48 ng/mL; Figure 1). After the exclusion of this outlier, a sex-specific difference could be detected (*p* = 0.0499). Serum Oc levels were significantly higher in female mice (54.20 [45.25; 59.01] ng/mL) compared to age-matched male mice (39.04 [35.18; 48.01] ng/mL) (*p* = 0.008) (Figure 1).

### 2.2. Trabecular Compartment

Table 1 shows the trabecular bone mineral density and microstructural parameters of the four evaluated regions. BV/TV was significantly higher in male than female mice. Compared to female mice, TbN was significantly higher and TbSp was significantly lower in male mice at all sites tested. TbTh and Tb density did not significantly differ by sex.

In female mice, we found no correlations between trabecular parameters at different sites. In male mice, positive correlations were detected between the femur and the tibia for BV/TV (r = 0.842; *p* = 0.004) and TbTh (r = 0.842; *p* = 0.004); between the femur and the lumbar spine for density (r = 0721; *p* = 0.023). BV/TV (r = 0.745; *p* = 0.017), TbTh (r = 0.830; *p* = 0.005), TbSp (r = 0.745; *p* = 0.017), and TbN (r = 0.721; *p* = 0.023) at the tibia were also correlated with the respective parameters at the lumbar spine. The structural parameters of the thoracic spine did not correlate with the structural parameters of any other region.

### 2.3. Cortical Compartment

The CtTh of the femur was lower in males compared to female mice. The other cortical microstructural parameters did not differ between female and male mice (Table 2).

### 2.4. Sex-Specific Discrimination

**Figure 2:**
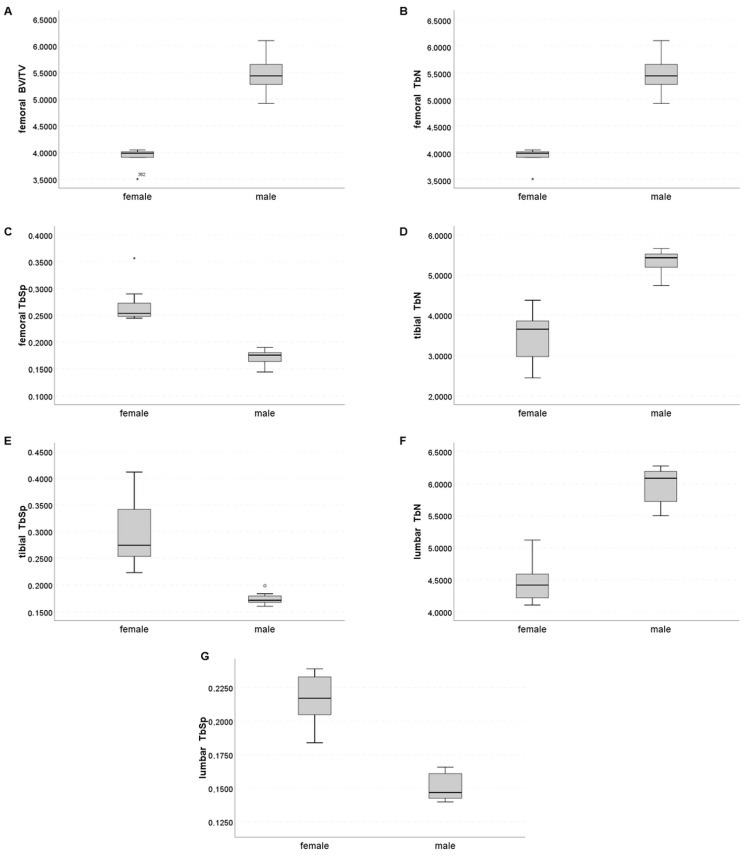
Boxplots of (**A**) femoral BV/TV, (**B**) femoral TbN, (**C**) femoral TbSp, (**D**) tibial TbN, (**E**) tibial TbSp, (**F**) lumbar TbN, and (**G**) lumbar TbSp. *, ° indicate outliers.

At the distal femur, three trabecular parameters (BV/TV, TbN, and TbSp) showed excellent performance for sex-specific discrimination (AUC 1.000). At the tibia and lumbar vertebral bodies, the AUCs for TbN and TbSp were also 1.000. The boxplots (Figure 2) show that there was no overlap between the lowest value of one group and the highest value of the other group for these values. Concerning the cortical compartment, femoral CtTh was the best parameter for sex-specific differentiation, with an AUC of 0.914 (Table 3).

## 3. Discussion

Despite the widespread use of mice in osteoporosis research, the literature contains relatively little data on sex-specific differences, and no recommendation exists on which bone site(s) and which microstructural parameter(s) are best to be evaluated. In this study, by using 6-month-old C57BL/6J mice, the most extensively used mouse strain characterized by a low bone mass phenotype, we showed sex-specific differences in microstructural parameters, which were mainly detected at the trabecular compartment of the distal femur, the proximal tibia, and the lumbar vertebral body. We further demonstrated that it is possible to discriminate male from female bone by assessing a single microstructural parameter at even only one skeletal site.

To relate differences in bone microarchitecture to bone remodeling, a bone resorption and formation marker were determined in the serum. Overall, female mice had higher serum levels of Oc and CTX than their male counterparts. In a previous study, 14-week-old female mice had significantly lower serum levels of CTX than male mice, but no differences were detected between the sexes in Oc [17]. Although no group comparison was performed in a study by Wejheden et al. [18], serum CTX levels in their study were lower than CTX levels in our investigation. These discrepancies may be explained by the use of different kits and differences in the animals’ ages. The C57BL/6 mice reach skeletal maturity before the age of 6 months [19], and BV/TV at the distal femur peaks even as early as 6–8 weeks, declining thereafter [13]. Thus, we assume that female mice have a higher bone turnover at the age of 6 months than male mice. This higher bone turnover is in accordance with the steeper decrease in BV/TV in female C57BL/6J mice shown by Glatt et al. [13], confirming that bone turnover markers (BTMs) can be used as surrogate markers for laborious histomorphometric analyses. Recently, Mun and coworkers [20] showed a higher osteoclast number at the femur of female C57BL/6J mice 8 weeks of age compared to age-matched male mice. They also demonstrated a sexual dimorphism in osteoclast differentiation with accelerated osteoclast differentiation of osteoclast precursors in female mice. These findings support our results showing higher serum CTX levels in female animals.

Microstructural analyses of trabecular bone revealed that, overall, BV/TV was significantly higher in male than female mice. In all bones assessed in male mice, TbN was higher and TbSp was lower than in the corresponding female bones. These significant sex-specific differences in trabecular microstructure are in line with previous animal studies [13,16,17,21]. For example, Mohan and coauthors [16] found lower BV/TV, TbN, and TbTh, but a higher TbSp in female femur metaphyses compared to femurs derived from male C57BL/6J mice at 16 weeks of age. In another study, utilizing C57BL/6 mice, female animals had significantly lower TbN at the femoral and tibial metaphyseal regions compared to male animals [17]. An investigation of hamsters detected similar sex-specific differences in these microstructural parameters; however, differences did not reach statistical significance before the age of 12 months [22]. In contrast to these and our results, one study using high-resolution DXA rather than µCT showed that male NMRI mice had a lower TbTh in the distal femur than their female counterparts [15]. The more favorable trabecular microstructure in males in our investigation may be due to the lower bone turnover rate in male C57BL/6J mice shown previously [23]. It is also in line with human studies that used high-resolution peripheral computer tomography (HR-pQCT) to evaluate the distal radius and the tibia in individuals aged between 15 and 90 years [3,4,5,6,7,8,24]. While evaluating cadaver donors, Chen et al. [25] detected significantly higher TbN in the lumbar spine of men (age range: 57–98 years) and increased BV/TV in the L4 vertebral body.

We detected less pronounced sex-specific differences in the cortical compartment. The CtTh of the femur was the only parameter differing by sex. The lower CtTh in male mice contrasts with human HR-pQCT studies [4,5,7,8] and may be explained by the fact that mice are quadrupeds. Thus, the stress on their femurs during locomotion is different from that in humans (i.e., who are bipeds). In mice, two studies [15,17] presenting data on femoral CtTh did not detect any sex-specific differences; one study [13] showed higher CtTh in female mice.

The microstructural differences between male and female skeletons in humans and animals led to the question of which microstructural parameters are best to distinguish male from female bone in mouse studies exploiting μCT technology. This study showed that each of the following trabecular microstructural parameters was able to distinguish 100% male from female bone: BV/TV (femur), TbN (femur, tibia, and lumbar vertebral body), and TbSp (femur, tibia, and lumbar vertebral body). Sex-specific differentiation of bones based on microstructural parameters of the cortical bone compartment proved more challenging in our study, with CtTh at the diaphysis of the femur performing best with an AUC of 0.914. Thus, collectively, our results suggest that the femur may be the best site for detecting sex differences in six-month-old C57BL/6J mice.

Another question of this study was: Is it meaningful to evaluate more than one bone site? In male mice, we detected correlations between trabecular bone microstructural parameters at different skeletal sites. These results confirmed the knowledge that microstructural parameters are heterogeneous [24], and site-specific, thus, there are limitations in drawing conclusions based on assessments of a single bone site. We suggest that if microstructure is important for the research question, different bones should be evaluated. Conversely, if the aim of the study is to evaluate sex differences, an assessment of bone microstructure in the femur, the tibia, or the lumbar spine would be sufficient. Due to the lack of correlation of structural parameters of the thoracic spine with other regions and the inability to distinguish male from female bones in the thoracic spine, we do not recommend the use of this bone. A relatively high variance in BV/TV in male femurs of Efna4 knockout mice has been shown previously [26]. Our study also revealed a small difference between female and male BV/TV in the femur compared to other microstructural parameters evaluated (Figure 2). However, this parameter allows detecting sex-specific bone differences, while it is the strongest predictor of bone strength and stiffness [27]. Thus, for sample size calculation, taking both functional and biomechanical aspects into consideration, we suggest the use of femoral BV/TV.

The study has some limitations. Its cross-sectional design did not allow us to assess changes associated with growth or aging. Using in vivo μCT imaging, age-related changes in the microstructural properties could have been detected. However, we decided not to use in vivo μCT because such an examination makes anesthesia necessary and exposes animals to radiation. We have previously investigated the development of bone microstructure in female and male mice of different strains, including C57BL/6J mice, up to 24 weeks of age [14,28]. Another limitation is that we only investigated C57BL/6J mice. Therefore, it is not possible to generalize our results to different mouse strains. Nevertheless, this is the first study to analyze the bone microstructure of two regions of the vertebral column and the skull in addition to conventional assessments of the femur and the tibia. Another strength of our study design is that μCT scanning was performed as a percentage of the total length of long bones instead of scanning a standard number of slices, thereby, potential size differences of individual bones were taken into account.

## 4. Materials and Methods

### 4.1. Animals

Ten female and ten male C57BL/6J mice were obtained from the “Abteilung für Labortierkunde und Genetik”, Zentrum für Biomedizinische Forschung, Medizinische Universität Wien (MUW, Himberg, Austria) at the age of 8 weeks. At our animal facility (Department of Pathophysiology and Allergy Research, MUW), animals were maintained in groups of 2–6 mice per cage under a standard 12-h light-dark cycle and had unlimited access to drinking water and food (LASQCdiet^®^ Rod 16, Auto; LASvendi GmbH, Soest, Germany). We euthanized the animals by carbon dioxide inhalation at the age of 6 months. All procedures were performed in accordance with the national and institutional laws and regulations. According to the Austrian animal experimental law, approval by the Ethical Committee was not necessary for this type of study.

### 4.2. Tissue Collection

Blood samples were obtained by cardiac puncture immediately after euthanasia by CO_2_ inhalation. The blood samples were centrifuged at 2500× *g* for 10 min and stored at −70 °C until analysis for bone turnover markers. The C-terminal telopeptide of type I procollagen, or (CTX; RatLaps™, Immunodiagnostic Systems IDS, Boldon, UK), a marker of bone resorption, and osteocalcin (Oc) (MicroVue^TM^ Bone, Quidel, Athens, OH, USA) a marker indicative of bone formation, were assessed with enzyme-linked immunosorbent assays (ELISA) according to the manufacturers’ instructions [21,29,30].

We assessed five distinct skeletal sites. The rationale for their selection was based on available research and literature gaps. Thus, we evaluated the bone microstructure of the femur and the tibia, as these are frequently assessed sites in experimental studies. Given that trabecular microstructure has shown intervertebral variation both in humans [31] and in senescence-accelerated (SAMP6) mice [32], we also analyzed the bone microstructure of the thoracic and lumbar vertebral bodies. Finally, we included assessments of the skull, as this is a site of flat bones not evaluated in mice so far. Therefore, femora, tibiae, thoracic, and lumbar vertebral bodies, and skull bones (parietal bones) were prepared by being fixed in 4% formaldehyde for 24 h and subsequently kept in ethanol (70%) at 4 °C until microstructural analysis.

### 4.3. Analysis of Bone Microstructure

**Figure 3:**
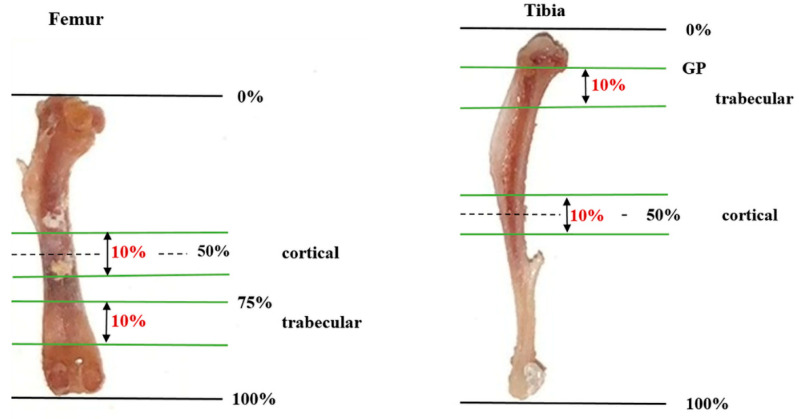
Schematic representation of the micro-CT scanning procedures for the assessment of bone microstructure at the distal femur and proximal tibia. The total bone length is displayed as 100%—proximal stated as 0%, distal bone as 100%. GP: growth plate.

The trabecular and cortical compartments of the aforementioned bones were analyzed by microcomputed tomography (μCT) using a microCT-35 device (Scanco Medical, Brüttisellen, Switzerland). The samples were placed in a sample-specific holder (20 mm in diameter) and turned into foam. The X-ray tube was operated at 70 kV with an intensity of 114 μA and an integration time of 800 ms, resulting in a resolution of 10 μm/pixel. Trabecular microarchitecture and density of the distal femur were assessed over 10% of the total bone length, starting at 75% of the overall length. Assessment of the trabecular microarchitecture of the proximal tibia started at the growth plate and was performed in the distal direction over 10% of the total length of the whole tibia. Femoral and tibial cortical morphometry were studied over a length of 5% proximal and 5% distal from the midline of the total bone length. Figure 3 shows a schematic representation of the micro-CT procedures followed for scanning the femur and the tibia. Vertebral trabecular microstructure was assessed at the thoracic vertebral body 12 (Th12) and lumbar vertebral body 5 (L5). The cortical morphometry of the skull (parietal bone) was evaluated with 232 slices performed behind the orbital towards the occipital bone. For the evaluation and reconstruction, the Xming™ program was used. The femur, tibia, vertebral bodies, and the skull were manually delineated. To separate bone from nonbone tissue, thresholds of 260 and 220 hydroxyapatite/cm^3^ (HA/cm^3^) were applied at the cortical and trabecular compartments, respectively.

Trabecular and cortical microstructural parameters were assessed according to the American Society for Bone and Mineral Research (ASBMR) guidelines [33]. The researcher who conducted this analysis was not aware whether the bones analyzed were from male or female animals. Trabecular parameters included trabecular bone volume fraction (BV/TV), trabecular mineral density (TbBMD), trabecular number (TbN), trabecular thickness (TbTh), and trabecular separation (TbSp), with the three latter parameters determined using the plate model (based on triangularization of surface). Distance transformation (without taking the shape of the bone into account) was the method used for the evaluation of cortical parameters: cortical bone area fraction (CtAr/Tt.Ar), average cortical thickness (CtTh), cortical porosity (CtPo), and cortical density (CtBMD). All selected parameters were automatically calculated by using the Xming™ program.

### 4.4. Statistical Analysis

All computations were performed using IBM SPSS Statistics for Windows version 26 (IBM, Armonk, NY, USA). Due to skewed data, metric data are described by the median [1st quartile, 3rd quartile]. In order to compare measures obtained from male and female mice, Mann–Whitney U-tests were used. ROC (receiver-operated characteristic) analyses and area under ROC curves (AUC) were calculated to assess the power of the obtained measures to detect sex differences. Spearman rank correlations were used to describe the linear association between trabecular measures. *p*-values ≤0.05 were considered statistically significant. Multiplicity correction (Bonferroni correction) was performed for trabecular as well as cortical parameters.

## 5. Conclusions

In this study, assessing sex differences in bone microstructure (by μCT) in 6-month-old C57BL/6J mice, we showed that BV/TV (femur), TbN (femur, tibia, lumbar spine), and TbSp (femur, tibia, lumbar spine) could be used to detect differences between sexes in the trabecular bone compartment. The CtTh at the distal femur was the best cortical microstructural parameter for discriminating male from female bone. In cases where it is not possible to assess multiple bone sites, we propose to evaluate the bone microstructure of the femur for detecting sex-specific differences. We also advise the use of femoral BV/TV for sample size considerations in future bone and osteoporosis research studies with C57BL/6J mice.

## Figures and Tables

**Table 1:** Trabecular bone mineral density and microarchitecture at four different skeletal sites in female (n = 10) and male (n = 10) C57BL/6J mice at 6-month of age.

	Femur			Tibia			Th12			L5		
	Female	Male	*p* Value	Female	Male	*p* Value	Female	Male	*p* Value	Female	Male	*p* Value
BV/TV (%)	0.162 [0.104; 0.199]	0.366 [0.285; 0.397]	0.0009	0.141 [0.113; 0.141]	0.282 [0.223; 0.306]	0.0040	0.362 [0.352; 0.380]	0.438 [0.423; 0.452]	0.0060	0.327 [0.313; 0.362]	0.413 [0.388; 0.461]	0.0060
TbN (mm^−1^)	3.99 [3.91; 4.03]	5.44 [5.25; 5.66]	0.0021	3.65 [2.89; 4.01]	5.43 [5.19; 5.54]	0.0004	5.15 [4.90; 5.34]	6.41 [6.27; 6.70]	0.0009	4.42 [4.20; 4.62]	6.08 [5.69; 6.19]	0.0009
TbTh (mm)	0.073 [0.060; 0.079]	0.083 [0.073; 0.088]	n.s.	0.067 [0.063; 0.070]	0.068 [0.061; 0.069]	n.s.	0.071 [0.067; 0.072]	0.067 [0.066; 0.070]	n.s.	0.070 [0.070; 0.071]	0.071 [0.064; 0.075]	n.s.
TbSp (mm)	0.254 [0.247; 0.281]	0.176 [0.164; 0.180]	0.0009	0.274 [0.246; 0.358]	0.171 [0.168; 0.181]	0.0004	0.178 [0.167; 0.187]	0.132 [0.126; 0.132]	0.0080	0.217 [0.202; 0.233]	0.147 [0.142; 0.161]	0.0009
TbBMD (mgHA/cm^3^)	651.0 [627.4; 686.2]	686.6 [658.4; 704.5]	n.s.	728.0 [706.9; 759.7]	716.1 [703.3; 730.4]	n.s.	676.2 [670.0; 703.9]	668.7 [646.0; 680.6]	n.s.	704.0 [684.6; 710.2]	698.3 [664.5; 717.3]	n.s.

Data are presented as medians [1st, 3rd quartiles]; Th12: thoracic vertebral body 12, L5: lumbar vertebral body 5; BV/TV: bone volume per tissue volume; TbN: trabecular number; TbTh: trabecular thickness; TbSp: trabecular separation; TbBMD: trabecular bone mineral density; *p*-values are given after multiplicity correction.

**Table 2:** Cortical bone mineral density and microarchitecture at three different skeletal sites in female (n = 10) and male (n = 10) C57BL/6J mice at 6-months of age.

	Femur			Tibia			Skull		
	Female	Male	*p*-Value	Female	Male	*p*-Value	Female	Male	*p*-Value
CtAr/TtAr (%)	96.85 [96.28; 97.21]	96.40 [96.66; 96.96]	n.s.	95.47 [94.32; 96.43]	96.56 [96.32; 96.84]	n.s.	82.64 [79.90; 83.84]	82.27 [80.21; 82.98]	n.s.
CtTh (mm)	0.21 [0.21; 0.22]	0.20 [0.20; 0.20]	0.0348	0.21 [0.20; 0.21]	0.21 [0.20; 0.22]	n.s.	0.11 [0.11; 0.12]	0.12 [0.12; 0.12]	n.s.
CtPo (%)	3.2 [2.8; 3.7]	3.6 [3.0; 4.3]	n.s.	4.5 [3.6; 5.7]	3.4 [3.2; 3.7]	n.s.	17.4 [16.2; 20.1]	17.7 [17.0; 19.8]	n.s.
CtBMD (mgHA/cm^3^)	992.5 [974.6; 999.1]	971.2 [949.4; 983.9]	n.s.	932.7 [904.4; 966.3]	960.5 [952.5; 967.3]	n.s.	717.5 [708.6; 725.0]	721.3 [709.4; 734.1]	n.s.

Data are presented as medians [quartiles]; CtAr/TtAr: cortical bone area fraction; CtTh: cortical thickness; CtPo: cortical porosity; CtBMD: cortical bone mineral density; *p*-values are given after multiplicity correction.

**Table 3:** Area under the curve of the trabecular and cortical compartments for all tested sites.

	AUC—Femur	AUC—Tibia	AUC—Th12	AUC—L5	AUC—Skull
**Trabecular compartment**					
BV/TV (%)	1.000	0.937	0.956	0.962	/
TbN (mm^−1^)	1.000	1.000	0.989	1.000	/
TbTh (mm)	0.800	0.522	0.689	0.656	/
TbSp (mm)	1.000	1.000	0989	1.000	/
TbBMD (mgHA/cm^3^)	0.743	0.633	0.717	0.625	/
**Cortical compartment**					
CtAr/TtAr (%)	0.729	0.839	/	/	0.570
CtTh (mm)	0.914	0.622	/	/	0.675
CtPo (%)	0.729	0.839	/	/	0.570
CtBMD (mgHA/cm^3^)	0.771	0.711	/	/	0.560

Th12: thoracic vertebral body 12; L5: lumbar vertebral body 5; BV/TV: bone volume per tissue volume; TbN: trabecular number; TbTh: trabecular thickness; TbSp: trabecular separation; TbBMD: trabecular bone mineral density; CtAr/TtAr: cortical bone area fraction; CtTh: cortical thickness; CtPor: cortical porosity; CtBMD: cortical bone mineral density.

## Data Availability

The data of this study are available from the corresponding author upon reasonable request.
